# Inter-Laboratory Validation of Method to Determine Residual Enrofloxacin in Chicken Meat

**DOI:** 10.1155/2018/6019549

**Published:** 2018-06-10

**Authors:** Joo Hee Chung, Kun Cho, Seongnyeon Kim, So Hyeon Jeon, Jeoung Hwa Shin, Jueun Lee, Yun Gyong Ahn

**Affiliations:** ^1^Seoul Center, Korea Basic Science Institute, Seoul 02841, Republic of Korea; ^2^Biomedical Omics Group, Korea Basic Science Institute, Ochang 28119, Republic of Korea; ^3^Western Seoul Center, Korea Basic Science Institute, Seoul 03759, Republic of Korea

## Abstract

An inter-laboratory study was performed to evaluate the performance of a method developed for the quantification of enrofloxacin in chicken meat. Liquid-liquid extraction combined with a clean-up procedure based on solid-phase extraction followed by a liquid chromatography-tandem mass spectrometric method was used by three individual laboratories. All the investigated results of calibration curves and limits of quantification were within the acceptable range for regulatory testing of enrofloxacin. The three laboratories received blind a certified reference material to analyze in triplicate and assess using statistical analysis. From the results, no statistical differences were found between the laboratories in the precision of the method. Additionally, all the results of the z-score, which is an indication of fixed interval bias criteria for accuracy from the laboratories, fell within the allowable limits (±2*σ*). Based on this proficiency testing by inter-laboratory comparisons, the analytical method including the sample preparation step was proven to be applicable.

## 1. Introduction

Enrofloxacin (ENR) is a fluoroquinolone (FQ) antibiotic that is a second-generation FQ modified from the quinolone family and commonly used in intensive poultry farming to treat chronic respiratory disease, colibacillosis, and fowl cholera. FQs have a large antibacterial spectrum of most Gram-positive and Gram-negative bacteria, and the mechanism of their action is performed by inhibiting bacterial DNA gyrase activity [1]. However, their long-term use results in residues in animal tissue followed by resistance to antibiotics. FQs also affect humans who consume food-producing animals and the environment [2]. Especially, imperfect elimination of* Campylobacter jejuni* from poultry's intestinal tracts by ENR can lead to survival of those bacteria and result in resistance to fluoroquinolone [3]. In 2005, the US Food and Drug Administration (FDA) stopped the use of ENR for bacterial infections in poultry and many countries have established maximum residue limits (MRLs) in their food products to safeguard human health from the risks of multiresistant and aggressive bacteria [4]. Monitoring studies of FQs include ENR residue in horse hair [5], muscle [6], intestinal tissue [7], milk [8], or chicken egg [9, 10], and they have been performed in accordance with related regulations because of resistance to the microbe in animal product consumers. Accordingly, there is a need for a validated analytical method as well as certified reference materials (CRMs) for the evaluation of such method. High performance liquid chromatography coupled with ultraviolet detect (HPLC-UV) [5], fluorescence detection (HPLC-FLD) [6, 8, 10], and mass spectrometry (LC-MS, MS/MS) [11, 12] have been used for quantitation. Among them, the use of LC-MS or LC-MS/MS is now preferred to the other methods because of the greater sensitivity and selectivity of FQs from the composite sample matrix. In addition, a CRM of ENR (KRISS CRM 108-03-003) has been developed recently by the Korea Research Institute of Standards and Science (KRISS) [12]. They has produced CRM samples that are bottled in 10 g portions in powder form, and its concentration has been measured by one point calibration with isotope-ratio matching [13].

The adoption of an analytical method can be officially approved on the basis of the results of a method's performance in inter-laboratory collaborative studies, and a comparison of these results is an external way of assuring quality control among laboratories concurrently. Inter-laboratory tests have been conducted to detect unsuspected errors and deficiencies in their methodology [14, 15]. Thus, in this study, three laboratories were involved in an inter-laboratory collaboration to test the evaluation method using a CRM. The same procedure, a liquid-liquid extraction combined with a clean-up procedure based on solid-phase extraction (SPE) followed by LC-MS/MS, was used by the three individual laboratories. In addition, linearity, limit of detection (LOD), and limit of quantification (LOQ) were each assessed, and the variance of results for the CRM between the laboratories was compared. The inter-laboratory comparison results showed that approximately 95 % of z-scores fell within two standard deviations (2*σ*), which is commonly designated as an acceptable range.

## 2. Materials and Methods

### 2.1. Chemicals and Reagents

CRM (No. 108-03-003, 10 g) for analysis of ENR in chicken meat was obtained from KRISS. ENR and enrofloxacin-d_5_ (hydrochloride salt form) as an internal standard were purchased from Dr. Ehrenstorfer GmbH (Augsbug, Germany) and Sigma-Aldrich (St. Louis, MO, USA), respectively. Acetonitrile, n-hexane, and methanol were purchased from J. T. Baker (Center Valley, PA, USA). The Oasis MAX (3 cc, 60 mg) was purchased from Waters (Milford, MA, USA), and the SupelMIP SPE-FQs (3 cc, 25 mg) were purchased from Sigma-Aldrich. Ammonium acetate and acetic acid were purchased from Fluka (Buchs, Switzerland). SAVANT SC210A SpeedVac (Thermo Fisher Scientific, San Jose, CA, USA) was used to evaporate the extract. Stock solutions (20 *μ*g/mL) for ENR and ENR-d_5_ were each prepared by dissolving them in acetonitrile. The working standard solutions were prepared at concentrations of 10, 20, 50, 100, and 200 ng/mL or 0.1, 0.5, 1, 2, 5, and 10 ng/mL depending on the instrument for each laboratory. The concentration of IS (ENR-d_5_) was 200 ng/mL in working standard. LOD and LOQ were estimated from calibration standards.

### 2.2. Preparation of Samples

**Figure 1:**
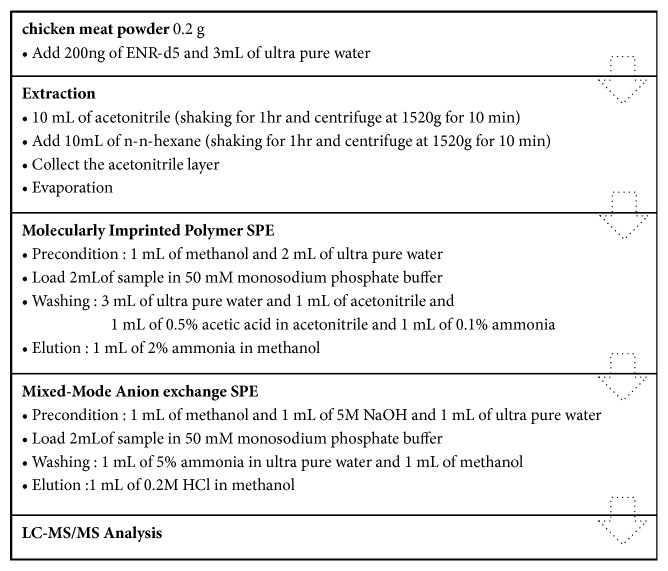
Analytical procedure for the determination of enrofloxacin in chicken meat.

Sample extraction procedure was modified from the recommended method by KRISS. Three laboratories took part in this study using the same procedure and a CRM with the same lot number. A 0.2 g amount of chicken powder was placed in a 50 mL conical tube and 100 *μ*L of ENR-d_5_ standard solution (2 *μ*g/mL) was added. Partitioning can separate ENR from impurities in a sample based on relative solubility [16]; hence a supernatant of the acetonitrile layer from water and a bottom layer from n-hexane were collected as shown in Figure 1. The dried extract was dissolved with 2 mL of 50 mM monosodium phosphate buffer (pH 7.4) for solubility enhancement of ENR [17] before application to the SPE clean-up. A molecularly imprinted polymer (MIP) known for its highly selective extraction of fluoroquinolones from food or environmental samples [18] was sequentially preconditioned with 1 mL of methanol and 2 mL of water. After the extract had been loaded onto the SPE cartridge, the cartridge was washed with water, acetonitrile, and 0.5% acetic acid in acetonitrile and then washed again with 0.1% ammonia solution. After drying up the washing solvent, 1 mL of 2% ammonia in methanol was eluted. To remove remaining interferences, the reconstituted eluent with 2 mL of 50 mM monosodium phosphate buffer was loaded onto a Mixed-Mode Anion exchange (MAX) SPE cartridge that had been preconditioned with methanol, 5 M NaOH solution, and water. After a washing step with 5% ammonia solution and methanol, 1 mL of 0.2 M HCl in methanol was eluted followed by drying under nitrogen. The dilution step is needed when the analyte concentration in reconstituted solution with 1 mL of acetonitrile exceeds the range of calibration or capability of the system. In the case of CRM sample, the solution was diluted by a factor of 10 because the positive ion electrospray (ESI+) exhibited a loss of detector response linearity. The overall analytical procedure of the sample preparation is described in more detail in Figure 1.

### 2.3. Analysis Using LC-MS/MS

Three types of LC-MS/MS systems were used as follows: Surveyor plus LC/TSQ Quantum ultra EMR system (Thermo Fisher Scientific, San Jose, CA, USA), 1290 Infinity II LC /6495 Triple quad MS system (Agilent Technologies Palo Alto, CA, USA), and 1200 LC/6460 Triple quad MS system (Agilent Technologies, Palo Alto, CA, USA). Chromatographic separation was achieved using a Zorbax Eclipse XDB-Phenyl column (Agilent, 150 x 3.0 mm, 3.5 um). The mobile phase was a binary mixture of 0.1% formic acid in water (A) and acetonitrile containing 0.1% formic acid (B) in a gradient elution mode at a flow rate of 300 *μ*L /min. The gradient elution profile was 10–90% (B) for 10 min, 90% (B) for 2 min, and 10% (B) for 8 min to condition and the injection volume is 10 *μ*L. The samples were analyzed in positive ion electrospray ionization mode with a spray voltage of 4 kV under an N_2_ sheath gas flow rate of 30 arbitrary units. The capillary temperature was maintained at 300°C. The multiple-reaction monitoring (MRM) transitions monitored were as follows: for ENR, 360.2 *m*/*z* → 316.2 *m*/*z* (quantifier ion) and 360.2 *m*/*z* → 342.2 *m*/*z* (qualifier ion); and for ENR-d_5_ (IS): 365.2 *m*/*z* → 321.2 *m*/*z* (quantifier ion) and 360.2 *m*/*z* → 347.2 *m*/*z* (qualifier ion).

## 3. Results and Discussion

### 3.1. Optimization of LC-MS/MS Condition

**Figure 2:**
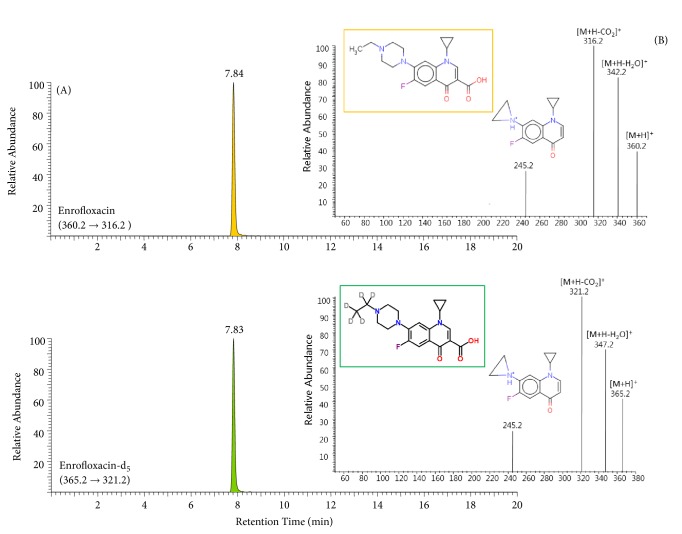
LC-ESI(+) MS/MS chromatogram (A) of enrofloxacin (upper panel) and enrofloxacin-d_5_ (lower panel) and each individual full-scan product ion spectrum (B) extracted from chicken meat CRM.

C_18_, phenyl, and pentafluorophenyl (PFP) reversed phase columns have been used to enhance the separation of ENR, which is an amphoteric substance with both acid and basic functionality, from biological samples [11, 12, 19]. While the separation of classical C_18_ phases is merely based on hydrophobic interaction between phase and analyte, phenyl type columns can offer selectivity to provide *π* -*π* interactions between the electron rich double bonds within the analyte and stationary phase phenyl moieties. Therefore, phenyl type columns were considered along with the selection of the mobile phase. According to Ferrari et al. (2015), ethylenediaminetetraacetic acid (EDTA 5 mM) was recommended as an organic modifier to avoid the result of peak tailing from strong interactions between ENR and the stationary phase when a PFP column is used [19]. Also, an analytical method related to a Zorbax Eclipse XDB-Phenyl column with mobile phase containing EDTA has been reported by Hyung et al. (2017)[12]. However, the use of EDTA turned out to be incompatible with continuous operation of an LC-ESI MS system, and this has been confirmed by other previous reports [20, 21]. In this study, without any tailing or excessive width, a peak for ENR was obtained using a Zorbax Eclipse XDB-Phenyl column with an EDTA buffer-free mobile phase (Figure 2(A)). The mass spectra of ENR and ENR-d_5_ each showed a protonated molecular ion [M+H]^+^ and two fragment ions ([M+H-H_2_O]^+^ and [M+H -CO_2_]^+^, in the positive ion mode. The fragment [M+H -CO_2_]^+^ ion undergoes further neutral losses (C_4_H_9_N and C_4_H_4_D_5_N) resulting in the formation of a common fragment ion, m/z 245 (Figure 2(B)). Two MRM transitions, a quantifier, and qualifier ion from each protonated molecular ion were used because this provides excellent sensitivity, selectivity, and speed.

### 3.2. Analytical Performance of Laboratories

Hyung et al. (2017) reported on the analytical procedures with CRMs for determining the FQ antibiotics in chicken meat [12]. They have been developing CRMs and making them available to analytical laboratories for the calibration of measuring instruments and evaluation of measurement methods or material properties. For a CRM of ENR in chicken meat (KRISS CRM 108-03-003), a certified value of 19.06 mg/kg with expanded uncertainty (95% confidence level) has been established by ID-LC/MS/MS measurements. However, it is rare to use only a one point calibration in GC or HPLC systems, and usually at least five to seven calibration points have been used to obtain accurate quantitative results [22, 23]. Also, validation parameters for linearity, LOD, and LOQ were not provided with their method.

However, an applicable analytical method with an LOQ at least below 30 ng/g is required in order to verify compliance with the Maximum Residue Limit (MRL) [24]. This study used an advanced method with appropriate determination of linearity and limit of quantification established through inter-laboratory studies; therefore, it is unique and distinctive from the work of Hyung et al. [12, 25]. LODs and LOQs can vary between analytical laboratories that have different instrumentation systems. For example, in a wide screening LC-MS/MS method by Love et al. (2012), the detection value and method detection limit ranged from 2 to 175 *μ*g/kg [26, 27] for the quantification of ENR; therefore, it is essential to have a clearly described procedure for estimating the LOD and LOQ during method validation to allow inter-laboratory comparisons.

The individual analytical performance parameters were undertaken by three participating laboratories as per ICH guidelines [28]. The linearity of the test method calibration over the concentration range of 0.1 to 200 ng/mL was confirmed by evaluating the regression coefficient-R^2^ according to each laboratory. All the investigated calibration curves were obtained by acceptable regression coefficients (R^2^ >0.99) and average response factors had a relative standard deviation (%RSD) less than 20. For the detection limits for LOD and LOQ, these values were adopted based on the standard deviation (SD) of the intersection of the analytical curve (s) and the slope of the curve (S) as LOD = 3.3 × (s/S) and LOQ = 10 × (s/S) and determined as shown in Table 1. It was established that all the results obtained by each laboratory satisfied the requirement for the MRL.

### 3.3. Inter-Laboratory Comparison of the Determination of CRM

Each bottle of CRM sample was prepared and analyzed in triplicate by the same analytical procedure at each of the three participating laboratories using an individual LC–Triple Quad MS/MS instrumentation system. An inter-laboratory comparison of the analytical results is presented in Table 2. The precision of the triplicate measurements per each laboratory was expressed as %RSD. For accuracy, bias was described as the difference between the average value of measurements per each laboratory and the average value of the CRM. One-factor analysis of variance was performed to determine whether there were any statistically differences in the means of the three participating laboratories for the results of the CRM. An *F*-Test was used to test the null hypothesis with raw data supported in Microsoft Excel 2013. The null hypothesis may be true, which means that there is no statistically difference, or alternatively the null hypothesis has to be rejected. Any difference in precision obtained from the CRM results was evaluated statistically using the *F*-test (Table 3), and the results for the three laboratories show that the *F* observed value (0.6161) is less than the *F* critical value (5.143), indicating that there are no statistically differences in precision obtained from the results between the laboratories. The null hypothesis cannot be rejected; in other words, no statistically significant difference was found in the results [29]. Moreover, results from the participants were used to calculate z-scores in accordance with the International Harmonized Protocol [30]. The z-scores compare the deviation of the results of each of the participants from the reference value with relative standard deviation (RSD) and can be calculated for the precision of the method, %RSD, in accordance with the Horwitz function.

**Figure 3:**
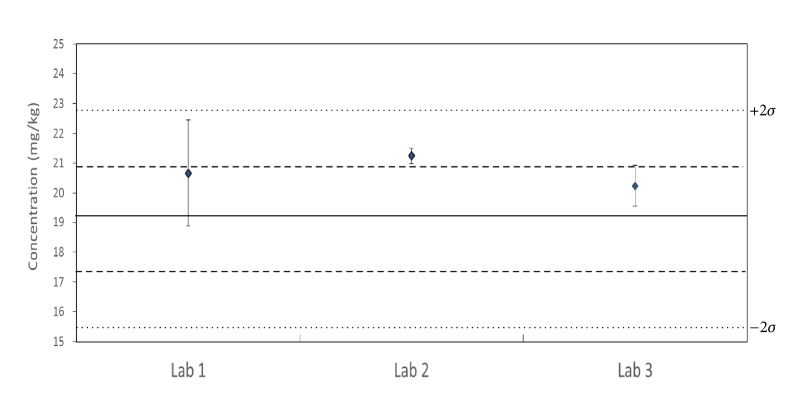
Interval of variation for CRM between three participating laboratories acceptance limits as *X*_*ref*_ ± 2*σ* (15.3 ~ 22.9 mg/Kg).

Based on the Horwitz formula (1), the RSD of reproducibility (%RSDR) was calculated as described in the following [31, 32]:

(1) $$\begin{array}{c} \left(\;\%\;\right)\;RSD\;Hortwitz=2^{\left(1-0.5logC\right)} \end{array}$$

where C is the concentration of analyte in dimensionless mass ratios. The standard deviation (*σ*) is used in the calculation of z-scores and provides scaling for laboratory deviation from the certified reference value. In the International Harmonized Protocol, assessment of z-scores is based on the following criteria: |z-score| ≤ 2.0 is regarded as satisfactory, 2.0 < |z-score| < 3.0 is regarded as a warning signal, and |z-score| ≥ 3.0 is regarded as unsatisfactory. The z-scores obtained by participating laboratories ranged from 0.62 to 1.2 and good agreement was found. Precision of the triplicate measurements between the three participating laboratories was evaluated using an individual value chart (Figure 3), and the three lines in the chart correspond to the reference value (solid line), *σ* (dashed line), and 2*σ* (dashed line) as acceptable limit. All laboratories fell within 2*σ*, a target criteria equivalent to 20% RSD based on the existing regulation.

## 4. Conclusion

An applicable analytical method for an MRL value to quantify ENR in chicken meat is a priority for regulation by a public health agency. Inter-laboratory comparison results as well as an improved analytical method for the quantification of ENR in chicken meat have been provided for regulatory testing. An appropriately determining linearity for quantification, LOD and LOQ, using an individual LC–Triple Quad MS/MS instrumentation system, has been established for three participating laboratories. From an inter-laboratory comparison of the determination of a CRM, accuracy and precision were assessed for the analytical method of each laboratory. There were no statistical differences for the results of the CRM between the participating laboratories by one-factor analysis of variance. Based on this proficiency testing by inter-laboratory comparisons, the analytical method including a sample preparation step was proven to be applicable. Furthermore, all laboratories obtained satisfactory z-scores that fell within 2*σ*.

## Figures and Tables

**Table 1:** Calibration curves and detection limits (LOD and LOQ) for individual laboratories.

Lab no.	Regression line				LOD (ng/mL)	LOQ (ng/mL)	Measured range (ng/mL)
	Slope	Intercept	R^2^	%RSD^1)^			
Lab 1	0.0045	0.0043	0.9996	8.05	7.28	21.8	10 ~ 200
Lab 2	0.010	−0.014	0.9990	2.63	0.39	1.17	0.1 ~ 10
Lab 3	0.024	0.014	0.9944	9.02	1.64	4.92	0.1 ~ 10

^1)^%RSD was calculated by average response factor (RF) for triplicate analysis.

*RF* = ((*Ax*)(*Cis*))/((*Ais*)(*Cx*)), where Ax is area of ENR; Cis is concentration of IS; Ais is Area of IS; Cx is concentration of ENR.

%*RSD* = (*Standard*  *Deviation*/*average*  *RF*)  *X*  100.

**Table 2:** Evaluation of precision, accuracy, and z-score between laboratories for CRM.

**Parameter**	**Lab 1**	**Lab 2**	**Lab 3**
Mean value, mg/kg	20.67	21.25	20.24
Number of sample, n	3	3	3
Precision			
Relative standard deviation (% RSD)	8.63	1.20	3.39
Accuracy			
Certified reference value, mg/kg	19.06 ± 0.86		
Bias, mg/kg	−1.61	−2.19	−1.18
Standard deviation (*σ*)^1)^	±1.91		
(10% RSD), mg/kg			
±2 *σ*, mg/kg	±3.81		
z-score^2)^	0.84	1.2	0.62

^1)^19.06 mg/kg as 10% RSD.

^2)^Z = (*x*_*lab*_ − *X*_*ref*_)/*σ*, where Z is z-score; *x*_*lab*_ is participant result; *X*_*ref*_ is certified reference value; *σ* os standard deviation.

|*z*| ≤ 2 is satisfactory.

2 < |*z*| < 3 is questionable.

|*z*| ≥ 3 is unsatisfactory.

**Table 3:** Statistical evaluation of the inter-laboratory measurements for CRM.

**Source of ** **Variation** ^**1)**^	**Sum of ** **Squares**	***df*** ^**5)**^	**Mean Sum of ** **Squares**	***F*** ^**8)**^	***p***	***F***_***crit***_^9)^
Between Laboratories	1.9396^2)^	2	0.9698^6)^	0.6161	0.503	5.143

Within Laboratories	7.5276^3)^	6	1.574^7)^			

Total	9.4672^4)^	8				

^1)^The output was performed in Microsoft Excel 2013.

^2)^Sum of squared deviations of group means from grand mean (SSB)

^3)^Sum of squared deviations of observations from their group mean (SSW)

^4)^Sum of squared deviations of observations from grand mean (TSS = SSB+SSW)

^5)^Degrees of freedom

^6)^ *SSB*/2

^7)^ *S* ^2^ = *SSW*/6

^8)^(*SSB*/2)/(*SSW*/6)

^9)^The critical value of *F* at 95% probability level is much higher (5.143) than the observed value of *F* (0.6161), which means that the null hypothesis cannot be rejected.

(The null hypothesis, which assumes that there is no difference between the data from three different laboratories)

## Data Availability

All relevant data are within the article and are available from the corresponding author upon request.
